# The Role of Healthcare Providers in Oral and Oropharyngeal Cancer Screening

**DOI:** 10.26502/fjhs.357

**Published:** 2025-10-21

**Authors:** Alexys Ferguson, Aaliyah Gray, Leela Subhashini Choudary Alluri, Krishna Patel, Monica Bean, Kandyce Kirt, Doney Eden, Lauren Jordan, Aleah Jackson, Samuel E. Adunyah, Pandu Gangula, James E. Cade, Zaid H. Khoury

**Affiliations:** 1 Department of Oral Diagnostic Sciences and Research, Meharry Medical College, School of Dentistry, Nashville, TN, 37208, United States; 2 Department of Periodontics, Meharry Medical College, School of Dentistry, Nashville, TN, 37208, United States; 3 General dentist, Rock Hills Dental Care, Hendersonville, TN, 37075 United States; 4 Department of Biochemistry, Cancer Biology, Neuroscience & Pharmacology, Meharry Medical College, School of Medicine, Nashville, TN, 37208, United States

**Keywords:** Carcinoma, Healthcare access, Healthcare quality, Leukoplakia, Mouth mucosa, Mouth neoplasms, Patient care team, Precancerous conditions, Quality of care

## Abstract

The oral cavity plays a vital role in the early detection and progression of systemic diseases, yet it is often underexamined by primary health care providers during medical evaluations. The global increase in both incidence and mortality rates of oral and oropharyngeal cancer (OC/OPC) highlights the urgent need for interprofessional collaboration of dentists and primary healthcare providers to mitigate this public health burden. Many at-risk patients are diagnosed at advanced stages, largely due to limited awareness among patients and healthcare providers, and a lack of affordability and accessibility to care. This results in a poor quality of life and, in some cases, death. The objective of this paper is to provide a comprehensive overview of head and neck examinations, introduce the application of a simplified oral cancer screening and referral form, and review common benign and oral potentially malignant disorders (OPMDs).

## Introduction

In recent years, the incidence and mortality rates of oral cancer (OC) and oropharyngeal cancer (OPC) have significantly risen globally and within the United States (U.S.) [1,2]. Mouth cancer is the 16^th^ most common cancer worldwide, with an estimate of 389,846 new cases in 2022 [3]. In the U.S., OC/OPC represents 2.9% of all new cancer cases, with an estimated 58,450 new cases in 2024 and 12,230 deaths [1]. The incidence, prevalence, and mortality rates of these forms of cancer vary across geographical regions and demographics with the majority occurring in Asia [4]. The most common type of OC/OPC is squamous cell carcinoma (SSC). The extent of SCC of the oral cavity proper involves the anterior two-thirds of the tongue, floor of the mouth, buccal mucosae, gingivae, labial mucosae, retromolar area and hard palate, with the most common location reported being the lateral borders of the tongue, floor of the mouth, and buccal mucosa. SCC of the oropharynx includes the base of tongue, soft palate, tonsils, and posterior pharyngeal wall and is usually diagnosed on the tonsils and base of the tongue. [5]. Common risk factors for SCC of the oral cavity proper include heavy tobacco with or without alcohol consumption, betel quid, Epstein-Barr virus, deficient diet, poor oral hygiene, immunosuppression, and previous cancer history. The human papillomavirus (HPV) infection, particularly high-risk variants such as HPV-16, plays a significant role in the onset of OPC, especially in younger patients, which may progress rapidly.[6] [7] In some instances, patients who are diagnosed with OC/OPC have little to no exposure to contributing risk factors.

OC/OPC is twice as common among males as females, with a higher prevalence in middle-aged White males. [7] Despite the advancements in early detection and treatment modalities, the overall 5-year survival rate remains poor (~69%) due to the lack of oral health accessibility, affordability, education, and late disease detection [1]. For Blacks, the 5-year survival rate is 52% compared to their White counterparts at 70% [2,8]. Educating healthcare providers on the proper techniques and landmarks of the oral cavity to perform thorough OC/OPC screenings can aid in early detection and intervention for many patients. When malignancies are identified in their incipient or premalignant stages, the 5-year survival rates often exceed 80% [1]. OC/OPC can be preceded by precursor lesions named oral potentially malignant disorders (OPMDs) [9]. OPMDs include leukoplakia, proliferative leukoplakia, erythroplakia, ulcers, and possibly oral lichen planus. While interviewing the patient, it is critical to pay special attention to presenting symptoms and the presence of established risk factors for OPMDs or OC/OPC [10]. Common symptoms or chief complaints include difficulty swallowing, a non-healing ulcer, abnormal growth of oral tissue, limited mouth opening, abnormal bleeding, a burning sensation when consuming acidic or spicy foods, and a lump on the neck in advanced cases. Surgical biopsy (excisional or incisional) remains the gold standard for diagnosing OPMDs and SSC, which is followed by histological grading, clinical staging, and management [11]. The objective of this paper is to provide a comprehensive overview of head and neck examinations, introduce the application of a simplified oral cancer screening and referral form, and review common benign and OPMDs.

## Clinical Assessment

**Figure 1:**
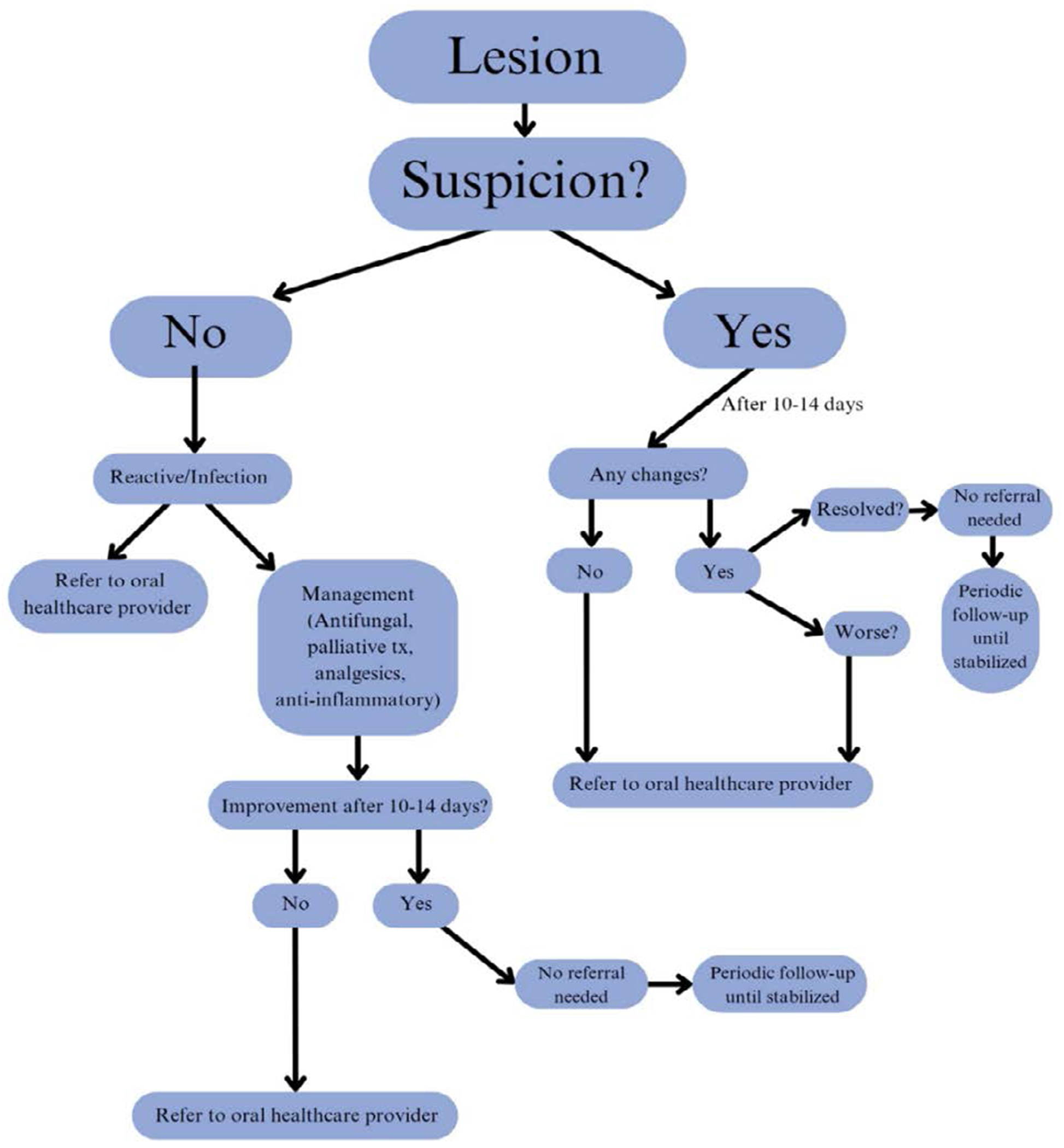
Schematic overview of decision-making for suspicious oral lesions. Oral healthcare providers include general dentists, OMS, and diagnostic specialists (e.g., OM).

**Figure 2:**
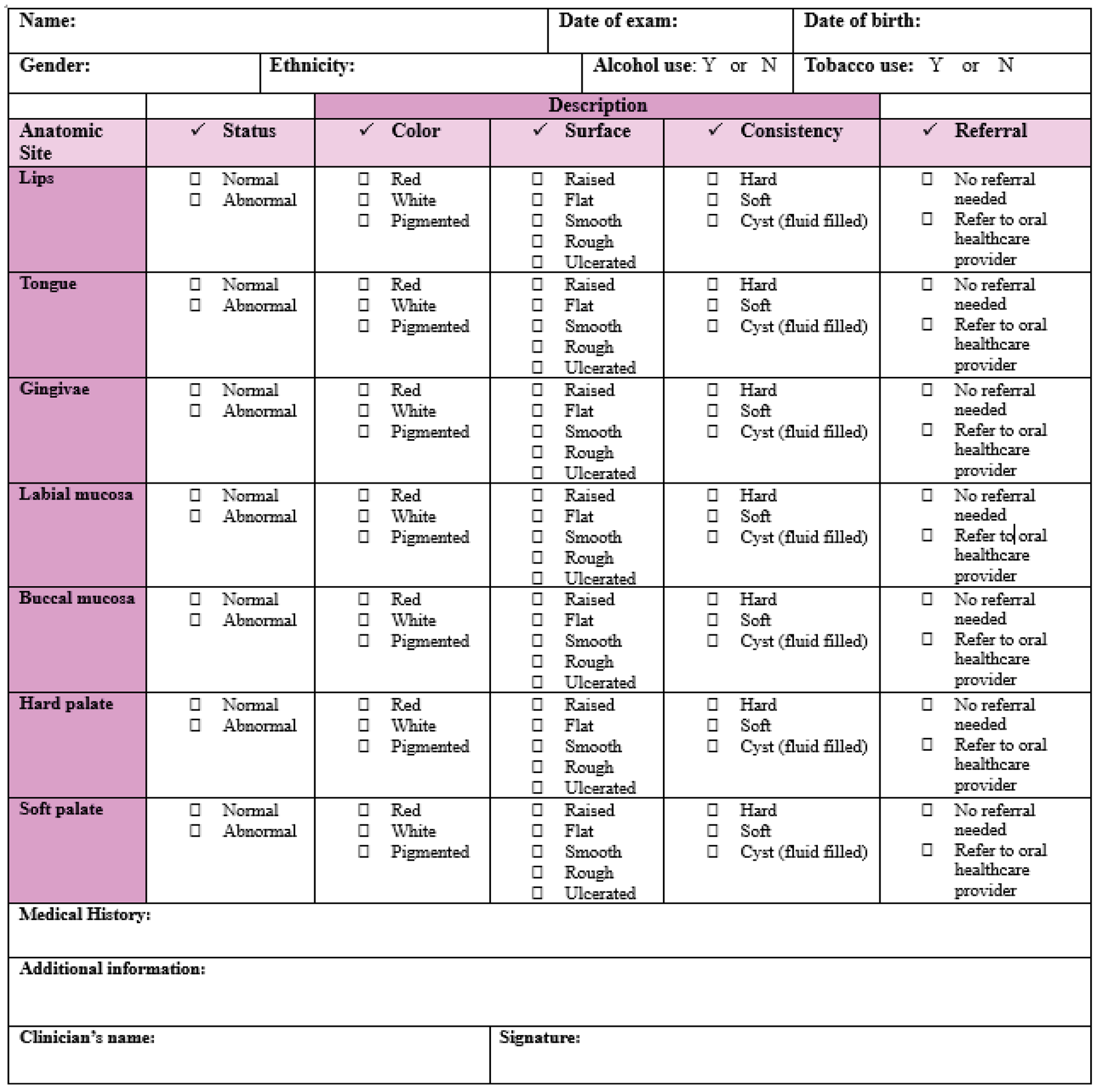
OC/OPC screening form. Referral to an oral healthcare provider includes a general dentist, OM, and OMS. Normal indicates no discoloration, lesions, pain, or bleeding present during the intra-oral and extra-oral examinations. Abnormal suggests discoloration, lesions, pain, and/or bleeding present.

According to the American Dental Association (ADA) guidelines, OC/OPC screening is a tool that involves using visual and tactile assessment of the head and neck extraoral and intraoral structures [12] [13]. Adequate lighting, examination gloves, personal protective equipment (PPE), a tongue depressor or disposable mouth mirror, and a piece of cotton gauze (2x2 or 4x4) can be used to perform this examination [14]. The integration of an OC/OPC screening form into routine medical examinations, combined with heightened awareness of high-risk clinical (“red flag”) signs associated with OPMDs and SCC (Table 1), may enhance the systematic, efficient, and comprehensive evaluation of patients. When clinical abnormalities or suspicious lesions are identified, primary healthcare providers should initiate appropriate management within their scope of practice or refer the patient to an oral healthcare specialist—such as a general dentist, oral and maxillofacial surgeon (OMS), or oral medicine (OM) specialist—for further evaluation, biopsy, and/or surgical intervention.Fig.1 represents a decision tree that healthcare providers can follow to ensure timely referrals when encountering a suspicious lesion. With the referral, a screening form, such as the proposed form in Fig 2 should be thoroughly completed and accompany the patient with intraoral and extraoral photographs, if taken. Certainly, photographs serve as valuable clinical documentation, providing oral healthcare providers with a visual reference of the lesion’s presentation at the time of examination, and must be included in the referral when feasible. Additional details such as the exact location of the lesion (maxillary or mandibular; left or right), the length of time in which the lesion was present, and the number of lesions should be included.

### Extra-Oral Examination

**Figure 3:**
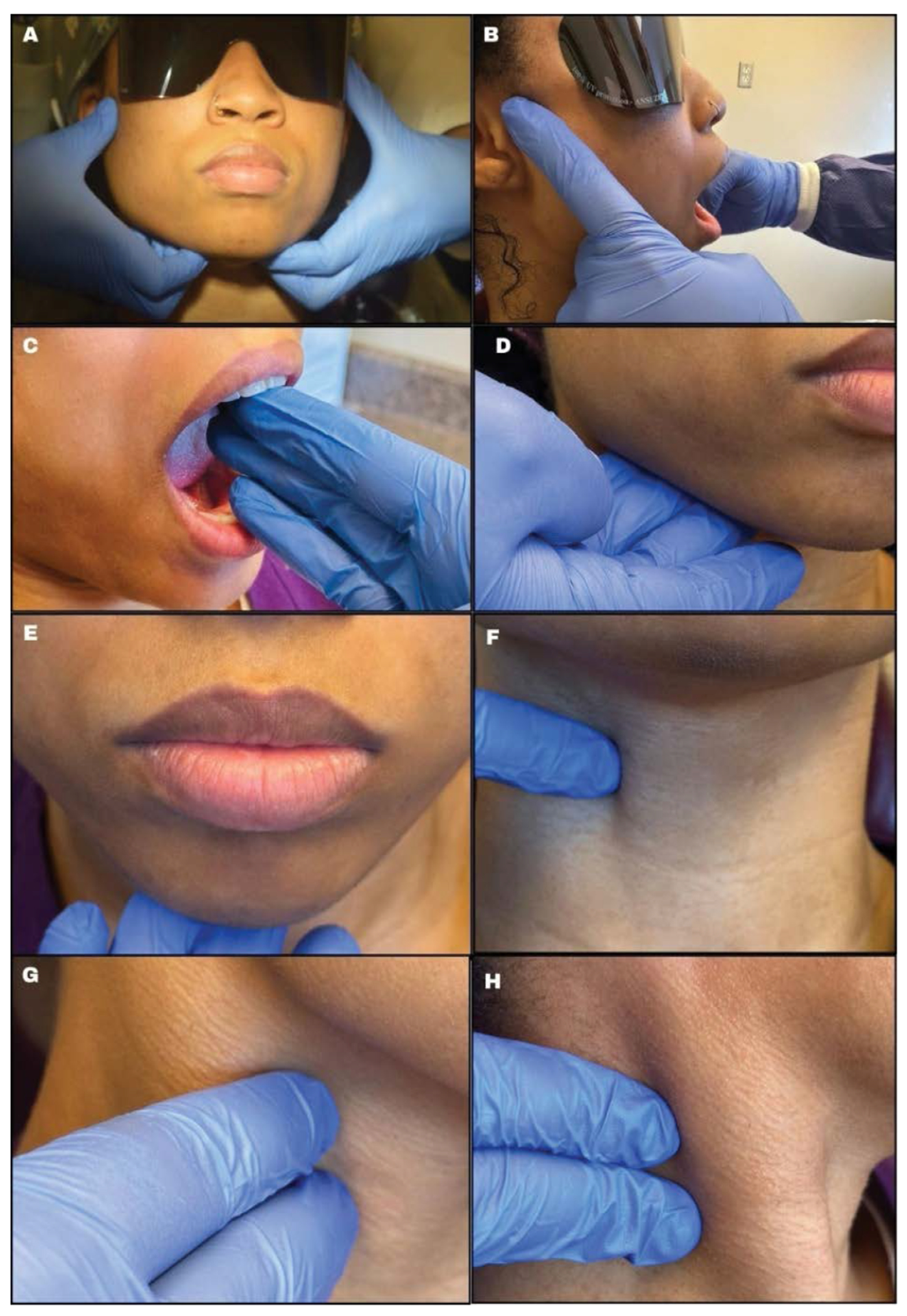
Extra-oral examination. (a) TMJ joint-bilaterally (b) Location of the lateral pole of right TMJ (c) Interincisal opening (d) Palpation of superficial submandibular lymph nodes (e) Palpation of the submental lymph nodes (f) Palpation of the thyroid gland (g) Palpation of deep cervical nodes (h) Palpation of the posterior triangle.

The healthcare provider should note any swelling, asymmetry, or surface changes during this examination [11]. The temporomandibular joint (TMJ) examination can be performed by standing behind (Fig. 3a) or in front (Fig. 3b) of the patient. With the use of clean gloves, the examiner’s thumbs should be placed anterior to the tragus of the ear, asking the patient to open and close slowly. The patient’s maximal opening can be examined by using three fingers (~47mm) to measure, as seen in Fig. 3c. [15] The importance of TMJ examination is to note any clicking, popping, crepitus (joint sounds), pain, limited mouth opening, or deviation of the mandible on either side. [14] Limited mouth opening, jaw, and ear pain can be a sign for patients living with advanced oral SCC [16].

**Figure 4:**
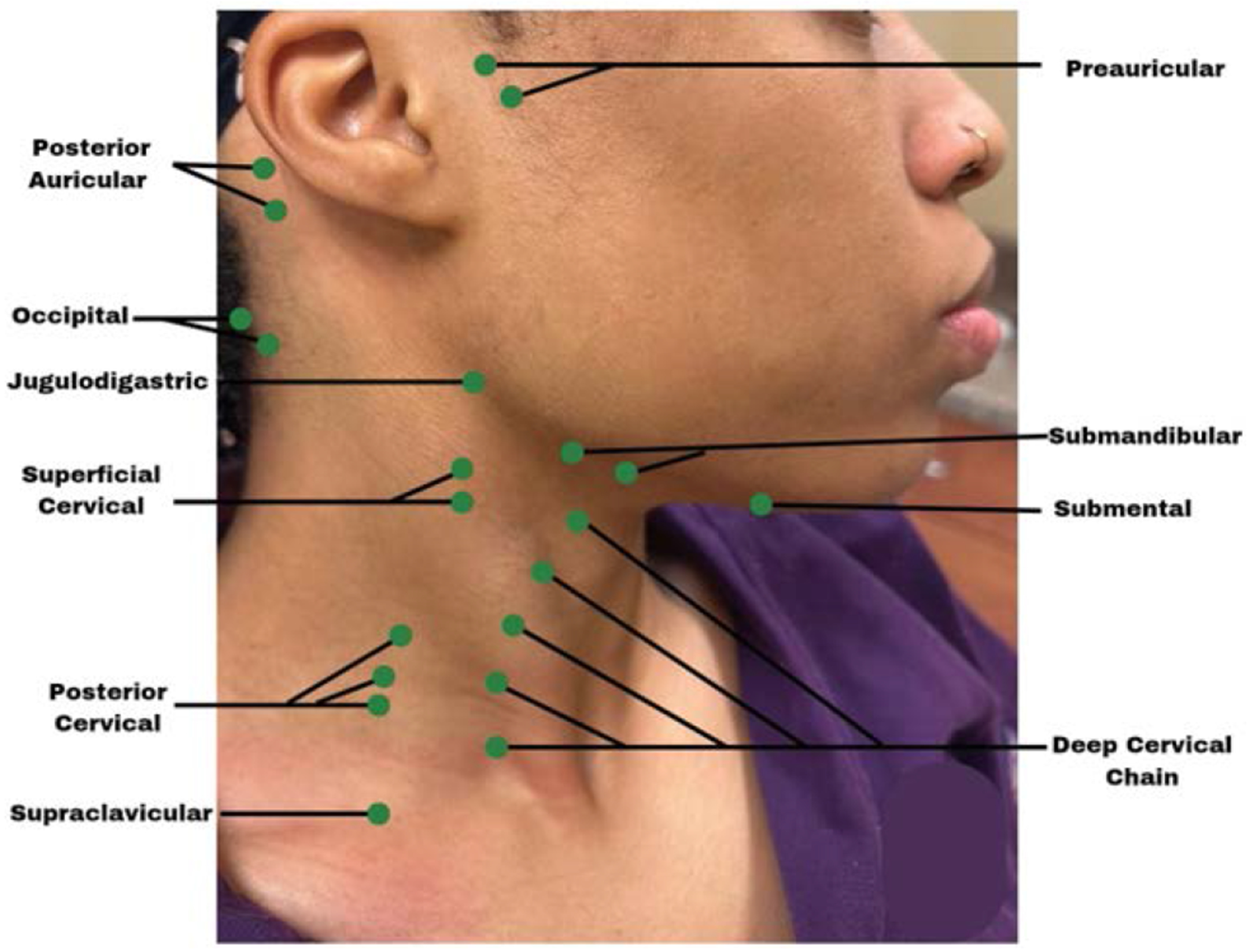
Lateral facial view highlighting the approximate anatomical location of head and neck lymph nodes, which are generally impalpable under normal conditions unless enlarged.

**Figure 5:**
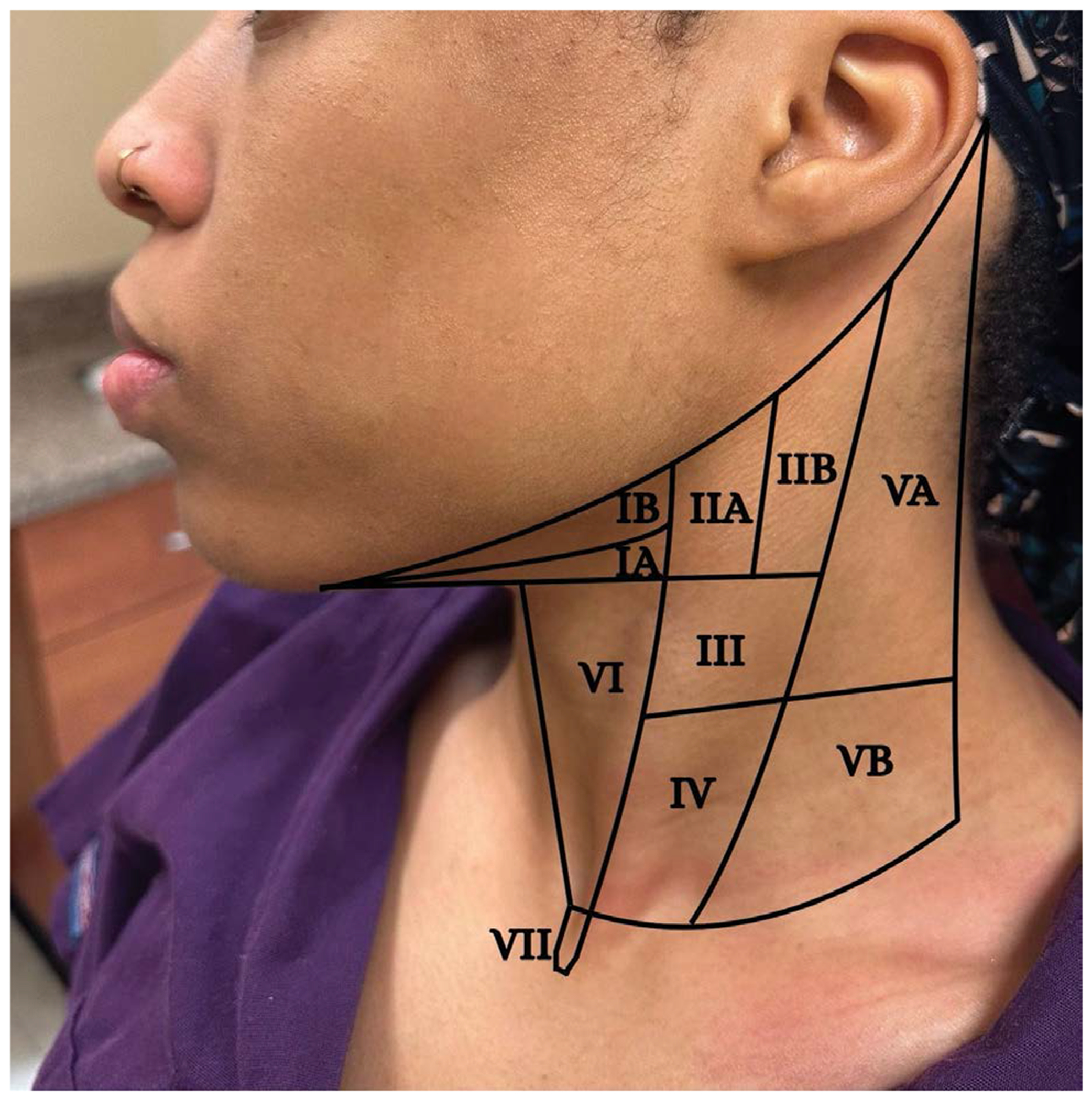
The seven levels of cervical lymph nodes.

Fig.3 d,e,g,h highlights tactile examination of the lymph nodes. Healthcare providers must understand the location (Fig. 4), drainage system, and the seven levels (Fig. 5) of the head and neck lymph nodes before examining the patient. During palpation, note any changes in size, texture (hard or soft), mobility (moveable or fixed), pain, or tenderness. An enlarged lymph node may result from inflammation, which could be infectious in origin, or neoplasia, or metastasis[17]. Acute inflammation mostly results in a tender and soft lymph node, which is freely movable from the surrounding tissue. Chronic inflammation, however, may result in a hardened lymph node. In contrast, metastasis often results in extracapsular tumor spread, which would manifest as matted lymph nodes or fixation of the lymph node to the surrounding soft tissues [17]. Patients might often complain about having a sore throat, “lump” in the throat or difficulty swallowing [16]. Metastasis to the head and neck lymph nodes usually occurs on levels I through III. OPC primarily affects levels II and III lymph nodes and may spread to the retropharyngeal nodes [18]. The spread of cancer cells in the head and neck lymph nodes can occur regionally, from the primary tumor site to a proximal lymph node, or distant, from the primary tumor site to distant parts of the body, such as the lungs, liver, and bones [18]. Examining the thyroid gland (Fig. 3f) can be done by standing in front of the patient, observing the neck midline for noticeable thyroid gland swellings (e.g. goiter) or asymmetry. Gently palpate the gland and record any nodules present [17].

### Intraoral Examination

**Figure 6:**
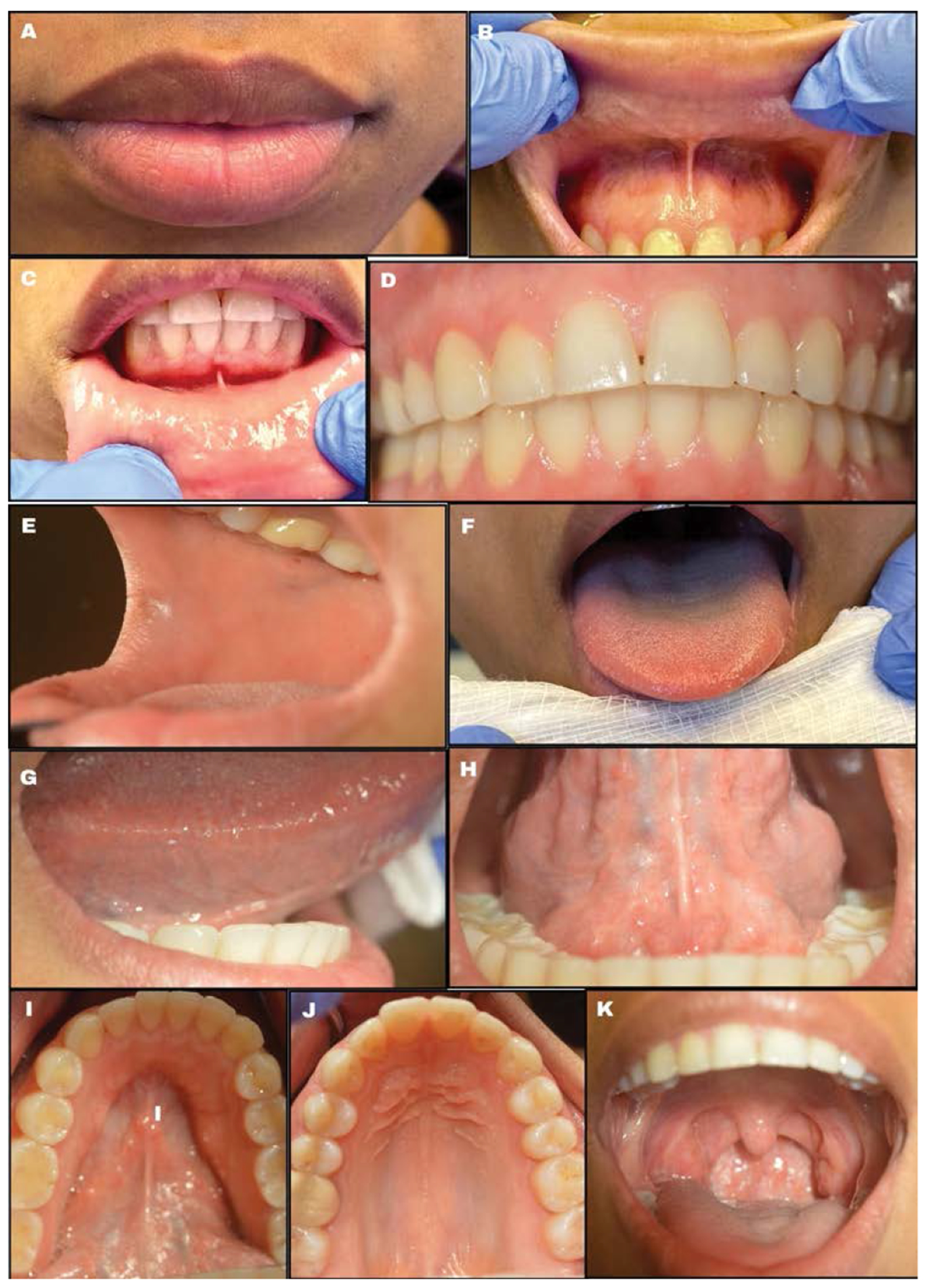
Intra-oral examination. (a) Lips (b) Upper labial mucosa/vestibule (c) Lower labial mucosa/vestibule (d) Gingivae (e) Buccal mucosa (f) Dorsal tongue (g) Lateral border of the tongue (h) Ventral surface of tongue (i) Floor of the mouth (j) Hard palate (k) Oropharynx.

**Figure 7:**
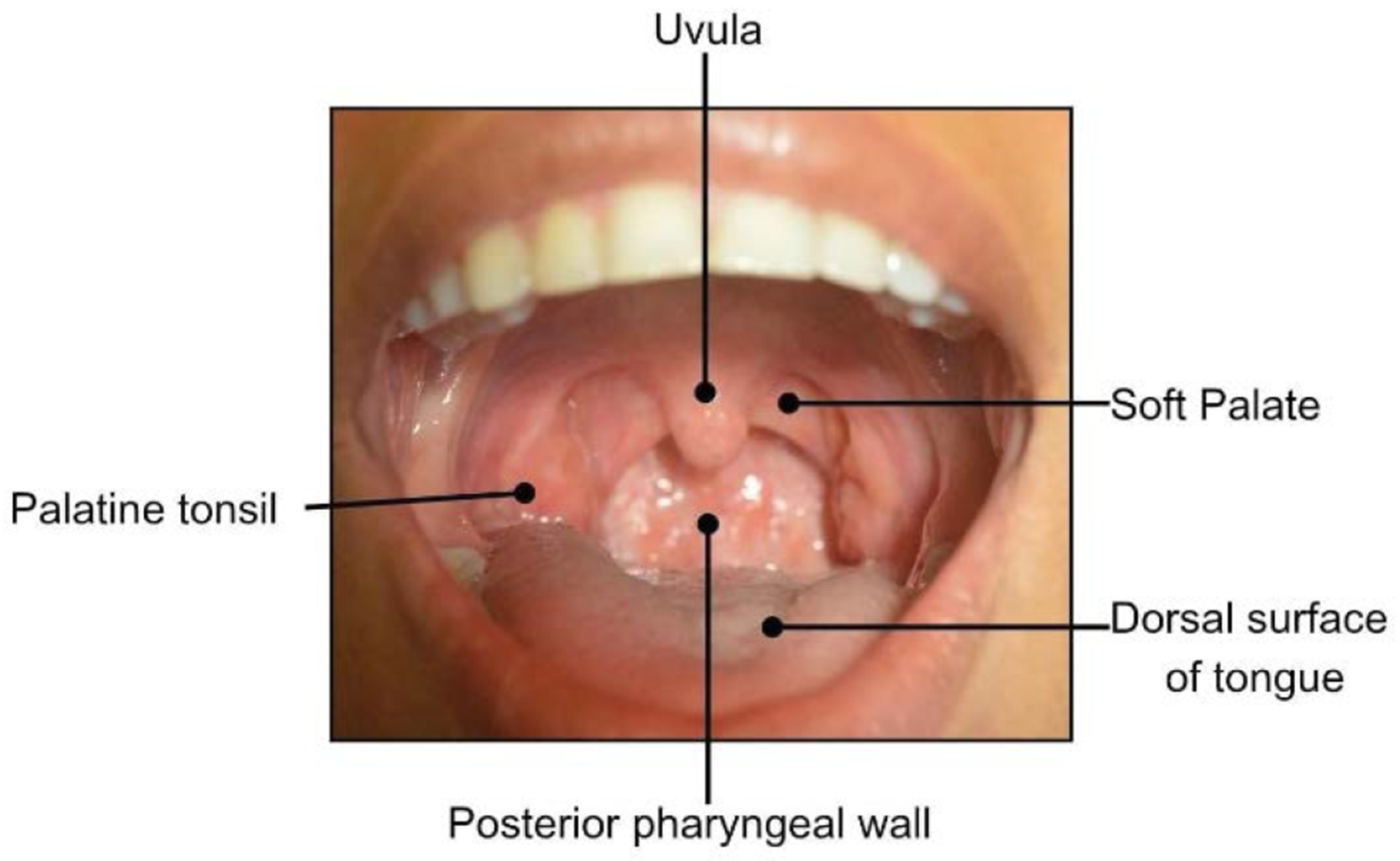
Labelling of the oropharynx

The healthcare provider should note any color (white, red, pigmented), texture (raised, flat, smooth, rough, ulcerated), or consistency (hard, soft, fluid-filled) changes during this examination. (Fig. 2). Lightly palpate the lips to determine if any lumps are present, which can indicate an infection or mucin spillage due to injury to minor salivary glands (e.g., mucocele) (Fig. 6a). Observe the texture of the lips. Increased sun exposure can cause blisters or non-healing ulcers (e.g., actinic cheilitis), which is a common risk factor for OSCC on the lips. Upper and lower labial mucosae (Fig. 6b-6c) can be examined using the thumb and index finger to reflect the lips upwards and downwards. Inspect the upper and lower labial mucosae. [17] Evaluate and palpate all gingival structures. Healthy gingival tissue is pink and stippled, as shown in Fig. 6d. Based on ethnicity, hyperpigmentation can be present as well. Further, the buccal mucosae can be examined by retracting the cheeks using a mouth mirror or tongue depressor to inspect and palpate the surrounding tissues (Fig. 6e). This is among the common sites for OC development. Using a 2x2 gauze the patient can be instructed to stick out their tongue and observe for any surface changes (Fig. 6f). With the same gauze, the tongue tip can be held and inspected/palpated bilaterally to ensure no lesions are present (Fig. 6g). The patient can then be instructed to lift their tongue to the roof of their mouth and inspect the underside of the tongue and floor of the mouth (Fig. 6h-i). This is a high-risk site for OC development. The hard palate can be visualized using a mouth mirror (indirect vision), and observed and palpated (Fig. 6j). The oropharynx can be examined also by using a mouth mirror or wooden tongue depressor, instruct the patient to open and say “ah” (Fig. 6k). The soft palate, tonsils, uvula, base of the tongue, and posterior pharyngeal wall must be inspected (Fig. 7) and any tonsillar exudate should be noted. This is a high-risk site for OPC development.

## Common Benign Oral Lesions

Primary healthcare providers are more likely to encounter benign pathoses as opposed to malignancies. Benign pathological processes are vast and encompass an array of developmental, reactive, traumatic, inflammatory (immune-mediated; infectious, noninfectious, or medication-induced), and neoplastic lesions (Table 2) [19]. Fordyce granules, palatal and mandibular tori, and fissured tongue are common developmental anomalies usually seen in adulthood [19]. Examples of reactive lesions include those caused by chronic friction, such as linea alba, morsicatio buccarum(chronic cheek biting), and fibrous hyperplasia [20]. Trauma can result in ulcerations and when minor salivary glands are involved, particularly of the lower lip, a mucocele (mucus-filled growth) can arise. Mucoceles are often seen in young adults and children but can occur at any age. [19]. Recurrent aphthous stomatitis, also known as canker sore, is an inflammatory, immune-mediated ulceration that is classified based on duration of onset, number of lesions, or underlying etiological factors into minor, major, and herpetiform variants. Factors that influence its onset include local trauma, stress, infection, and genetic predisposition [21]. Erythema areata migrans (geographic tongue) is common with an interplay of various underlying etiologies, mainly immune-mediated [22]. Infections, whether fungal (e.g., candidiasis (thrush), denture stomatitis, median rhomboid glossitis), bacterial (e.g., dental caries, periodontitis, actinomycosis), or viral (e.g., herpes labialis, squamous papilloma), can present with pain, fever, or malaise. [23] [22] Lipoma, hemangioma, and granular cell tumor, are examples of non-infectious, benign growths that are less likely to be encountered compared to reactive and infectious lesions. [24] [19] Hypersensitivities due to ingestion or topical placement of medications, usually present as a non-infectious, delayed cell-mediated reaction. Thermal and chemical burns are common as well, presenting as a superficial epithelial necrotic film, erythema to ulceration(s), associated with the site of contact or placement [25].

## Oral Potentially Malignant Disorders (OPMDs)

OPMDs are a group of lesions that carry an increased risk of malignant transformation and may precede the development of SSC [11]. Although OPMDs harbor a potential for malignant transformation, i.e., varying degrees of oral epithelial dysplasia, most do not progress to SCC [26].

### Leukoplakia

Oral leukoplakia (OL) is thought to be the most common OPMDs [27]. It’s a descriptive term for a white patch or plaque with well-defined borders that cannot be wiped off and is not attributable to any other cause (e.g., not caused by friction, *Candida albicans*, etc.) [26]. Like OC/OPC, factors contributing to the development of OL encompass various forms of tobacco use, alcohol consumption, and exposure to ultraviolet radiation when it involves the vermilion border. Other possible risk factors include fungal infections, viral infections, and hormonal imbalances [28].

### Proliferative Leukoplakia

Proliferative leukoplakia (PL) or proliferative verrucous leukoplakia (PVL) (when the surface is warty), represents a dynamic lesion with a significant potential for malignant transformation [29]. This distinct variant of OL predominantly affects females, with the gingivae and buccal mucosae being the most common sites [29] [30]. It may begin manifesting as a single innocuous lesion but often spread to multiple sites with a high probability of recurrence and malignant transformation, mainly into SCC [29].While the exact cause of PL remains uncertain and is usually not associated with risk factors of conventional OL, there is speculation in the literature about its potential association with HPV [29].

### Erythroplakia

The prevalence of erythroplakia is difficult to ascertain, likely due to the bright red color that is often obscured by the pink mucosal color, as well as heterogeneous and inconsistent global reported data [31]. Similar to OL, erythroplakia is a clinical descriptive term that should be diagnosed after excluding other recognizable conditions (e.g., vascular malformations, erythematous candidiasis). Clinically, it presents as a distinct red patch with well-defined borders and is often slightly depressed compared to the surrounding tissue [32]. Common sites for oral erythroplakia include the floor of the mouth, buccal mucosa, and soft palate. Etiological factors parallel those for conventional OL and include tobacco, betel quid, and alcohol [32]. Erythroplakia typically carries a higher malignant transformation rate compared to OL, with most biopsied erythroplakias presenting advanced oral epithelial dysplasia or SCC [31].

### Oral Submucous Fibrosis

Oral submucous fibrosis (OSMF) is a persistent and challenging condition affecting the oral cavity and occasionally extending to the pharynx [33]. It predominantly affects Southeast Asians and usually presents in adults aged 25 to 35 [34]. OSMF is linked to various causative factors, with the consumption of areca nut being a main contributor, along with deficiencies in micronutrients such as iron, zinc, and essential vitamins [33]. OSMF is characterized by an inflammatory reaction near the epithelium, followed by fibroelastic changes in the lamina propria layer. This results in epithelial atrophy, leading to stiffening of the oral mucosa due to extensive progressive fibrosis, ultimately causing trismus and difficulty in mouth opening [30].

### Ulcers

Ulcers of the oral mucosa are common, particularly in children, female teens and young adults [35] and are often associated with trauma, stressful events and hormonal changes [36]. An ulceration can also occur after consuming spicy or hot foods [37]. Ulcers lasting more than two weeks are considered chronic, while those lasting less than two weeks are acute and typically resolve on their own once the underlying causation is eliminated. [35] A non-healing chronic ulcer, particularly with rolled-out and indurated borders, can be a sign of SSC [35] and should be viewed with caution and referred to the appropriate oral healthcare providers for further management [36].

### Reverse-Smokers’ Palate

Palatal lesions known as smoker’s palate (nicotinic stomatitis) are predominantly found on the mucosa of the hard and soft palate, primarily among cigarette smokers, with a lesser occurrence among cigar smokers [38]. Those lesions result from heat irritation with mucosal changes that are often reversible upon stopping the habit [38]. Some users, however, place the cigarette in the opposite direction with the lit end facing the inside of the mouth. This habit may result in what is known as reverse-smokers’ palate, which presents as white and red mucosal changes that carry an increased risk of malignant transformation , due to the combined effect of heat and carcinogens [39]. It is also important to note that tobacco may induce mucosal hyperpigmentation as well, which may produce a mixed white, red, and brown lesion [39].

### Tobacco Keratosis

Smokeless tobacco users, such as those who chronically chew or use snuff, are at high risk of tobacco keratosis [40]. The lesion is greyish white in appearance and has a corrugated surface, and is located on the mucosal tissue of the area where the smokeless tobacco has contacted [40].The most common site for tobacco keratosis is in the lower anterior vestibule, but it is also frequently observed in the posterior vestibule.

### Plummer-Vinson Syndrome

Dysphagia, iron deficiency anemia, and esophageal webbing are the classic triad of a condition termed Plummer-Vinson syndrome (also known as Paterson-Kelly syndrome) [41]. It is mainly seen in middle-aged women and is associated with other syndromes such as gastrointestinal malabsorption conditions (e.g., celiac disease, Crohn’s disease), rheumatoid arthritis, and thyroid disease [42]. Iron supplements are given as part of the patient’s management. Plummer-Vinson syndrome can carry an increased risk for SCC of the pharynx and esophagus [42].

## Oral Lichen Planus

The malignant potential of oral lichen planus (OLP) has been debated, with variable reports regarding the risk of malignancy. OLP is an idiopathic, chronic, immune-mediated inflammatory disease that affects the skin and mucous membranes of the oral cavity [43]. OLP primarily affects middle-aged females and presents as an asymptomatic lace-like network of white striae (Wickham striae), typically bilaterally on the buccal mucosae (reticular type), and less commonly, as symptomatic erosive/ulcerative lesions of the oral mucosae (erosive type) [44]. As opposed to the reticular type, the erosive type of OLP is thought to carry an increased risk of malignant transformation. The primary treatment options for symptomatic cases include corticosteroids, retinoids, calcineurin inhibitors, and natural alternatives [45].

## Oral and Oropharyngeal Cancer and Management

The clinical presentation of SCC of the oral cavity is variable and may be preceded by an OPMDs, such as OL, or may arise de novo [26]. Clinically, oral SCC may be presented as a flat white, red, or speckled lesion with variable surface architectures, a necrotic ulcer with rolled-out borders that may invade neighboring anatomical structures, or a polypoid mass. The clinical presentation for oropharyngeal SCC is typically of redness in the posterior oral cavity/tonsillar area that is often accompanied by soreness or a feeling of a “lump” during swallowing and speech [26]. In a subset of oropharyngeal carcinomas, the primary tumor cannot be assessed, but rather it presents as a mass in cervical lymph nodes, producing palpable lymph node(s) [46]. Clinical staging is of paramount importance to determine the prognosis of OC/OPC. The management of OC/OPC requires a collaborative interdisciplinary team [46]. Therapeutic modalities range from surgery with or without neck dissection, radiotherapy, chemotherapy, and, more recently, immunotherapy [47]. The prognosis of OPC is generally better than OC. This is due to the enhanced responsiveness of OPC to chemoradiation as opposed to OC, limiting the need for invasive surgery [48]. The reason for this enhanced responsiveness is attributed to HPV-associated inactivation of tumor suppressor genes rather than mutating them, as seen in the carcinogenesis of OC [48].

## Advancements in Early Detection of OPMDs and OC/OPC

Several adjunctive diagnostic tools—such as brush biopsies, toluidine blue staining, and tissue autofluorescence—have been developed to support clinicians in the early detection of OPMDs and SCC. However, these modalities are often limited by factors such as increased procedural time, higher costs, patient discomfort, and suboptimal diagnostic accuracy, including the risk of both false-positive and false-negative outcomes.[49] Studies suggest that the primary limitation of these technologies lies in their low specificity for detecting dysplasia, which may lead to unnecessary biopsies in cases with coexisting inflammation. Conversely, false-negative results may occur in lesions exhibiting hyperkeratosis, potentially delaying definitive diagnosis [50].

Currently, the most common diagnostic devices for inoffice use are light-based systems using autofluorescence (e.g. OralID and VELscope^®^). These Food and Drug Administration (FDA) approved devices are user-friendly and are based on the principle that abnormal metabolic or structural changes exhibit distinct absorbance and reflectance autofluorescence properties [49,51]. This yields the emission of green autofluorescence imaging for normal tissues, whereas the affected tissue is an irregular, dark area that stands out [49] [51]. Although these adjunctive techniques may serve as an aid to the healthcare provider in screening for suspicious lesions, a surgical biopsy is still required for accurate diagnosis. Certainly, the initial biopsy should be performed with a surgical stainless-steel blade and submitted in 10% neutral buffered formalin or a suitable alternative. Artificial Intelligence (AI) has gained popularity in dentistry as a diagnostic aid due to its capability in identifying abnormalities that might go unnoticed by the untrained human eye [52]. The three crucial steps for implementing AI in diagnostic imaging are preprocessing, image segmentation, and postprocessing [53]. In a recent study done by Song et al., a low-cost, dual-mode, smartphone screening device was used to capture intra-oral images of patients from low and middle-socioeconomic communities living with suspicious oral lesions [54]. Despite some limitations, this device was able to distinguish between dysplasia and malignancy versus normality in 170 images with an average accuracy of 86.9%, sensitivity of 85%, and specificity of 88.7% [54]. Combining AI with other noninvasive methods shows promising outcomes for healthcare providers in confirming diagnoses in the future.

## Conclusions

This manuscript highlights the essential role of primary healthcare providers in the early detection and screening of OPMDs and SCC. Timely recognition and intervention can enhance patient outcomes through multidisciplinary collaboration. Primary care providers must remain vigilant to the clinical signs and symptoms of OPMDs and OC/OPC. The integration of standardized screening protocols and adjunctive diagnostic tools into routine clinical practice may facilitate earlier referrals and more effective management. Future research should prioritize the validation and implementation of the proposed screening and referral protocols to strengthen continuity of care within an integrated healthcare system.

## Figures and Tables

**Table 1:** Common OC/OPC high-risk clinical signs, “red flags”.

Screening key points	“Red flags” list
1. Collect medical and social history	✔ Tobacco use (amount, frequency, duration)
	✔ Alcohol use (amount, frequency, duration)
	✔ HPV status (vaccination)
	✔ Cancer history
	✔ Occupation (chronic sun exposure “cracked” or “blistered”)
2. Perform extraoral examination	✔ Limited mouth opening
	✔ Swollen lymph nodes
	✔ Facial asymmetry
	✔ Blisters on the face or lips (duration)
3. Perform intraoral examination	✔ Red, white or pigmented lesions on soft tissue (e.g. friction, burn, ill-fitting denture)
	✔ Texture changes (raised, flat, smooth, rough, ulcerated)
	✔ Pain during swallowing (“lump” in throat)
	✔ Poor oral hygiene (calculus, mobile teeth, exudate)
	✔ Bleeding
	✔ Numbness
4. Referral (if needed)	✔ Oral healthcare providers

**Table 2:** Overview of common benign oral lesions

Common Benign Oral Lesions	
*Type*	*Examples*
1. Developmental	Fordyce granules, palatal and mandibular tori, fissured tongue
2. Reactive	Linea alba, chronic cheek biting, fibrous hyperplasia
3. Trauma	Mucocele, traumatic ulceration
4. Inflammatory	
a. Immune-mediated	Geographic tongue, canker sore
b. Infectious	
i. Fungal	Thrush, denture stomatitis, median rhomboid glossitis
ii. Bacterial	Dental caries, periodontitis, actinomycosis
iii. Viral	Herpes labialis (cold sore), squamous papilloma
5. Medication-induced	Chemical burn, hyperpigmentation
6. Benign neoplasms	Lipoma, hemangioma, granular cell tumor

## Abbreviations

OC: Oral cancer
OPC: Oropharyngeal cancer
HPV: Human Papillomavirus
ADA: American Dental Association
US: United States
OPMDs: Oral potentially malignant disorders
SSC: Squamous cell carcinoma
TMJ: Temporomandibular joint
OMS: Oral maxillofacial surgeon
OM: Oral medicine
PPE: Personal protective equipment
OL: Oral leukoplakia
PL: Proliferative leukoplakia
PVL: Proliferative verrucous leukoplakia
OSMF: Oral submucous fibrosis
OLP: Oral lichen planus
FDA: Food and Drug Administration
AI: Artificial intelligence
